# Evaluation and Improvement of Image Aesthetics Quality via Composition and Similarity

**DOI:** 10.3390/s25185919

**Published:** 2025-09-22

**Authors:** Xinyu Cui, Guoqing Tu, Guoying Wang, Senjun Zhang, Lufeng Mo

**Affiliations:** 1 College of Software, Quanzhou University of Information Engineering, Quanzhou 362000, China; cuixy@stu.zafu.edu.cn; 2 College of Mathematics and Computer Science, Zhejiang A&F University, Hangzhou 311300, China; 20100075@zafu.edu.cn (G.T.);; 3 Shaoxing Institute of Technology, Shaoxing 312000, China; 4 Information and Education Technology Center, Zhejiang A&F University, Hangzhou 311300, China

**Keywords:** computational aesthetics, semantic segmentation, image retargeting, composition optimization

## Abstract

The evaluation and enhancement of image aesthetics play a pivotal role in the development of visual media, impacting fields including photography, design, and computer vision. Composition, a key factor shaping visual aesthetics, significantly influences an image’s vividness and expressiveness. However, existing image optimization methods face practical challenges: compression-induced distortion, imprecise object extraction, and cropping-caused unnatural proportions or content loss. To tackle these issues, this paper proposes an image aesthetic evaluation with composition and similarity (IACS) method that harmonizes composition aesthetics and image similarity through a unified function. When evaluating composition aesthetics, the method calculates the distance between the main semantic line (or salient object) and the nearest rule-of-thirds line or central line. For images featuring prominent semantic lines, a modified Hough transform is utilized to detect the main semantic line, while for images containing salient objects, a salient object detection method based on luminance channel salience features (LCSF) is applied to determine the salient object region. In evaluating similarity, edge similarity measured by the Canny operator is combined with the structural similarity index (SSIM). Furthermore, we introduce a Framework for Image Aesthetic Evaluation with Composition and Similarity-Based Optimization (FIACSO), which uses semantic segmentation and generative adversarial networks (GANs) to optimize composition while preserving the original content. Compared with prior approaches, the proposed method improves both the aesthetic appeal and fidelity of optimized images. Subjective evaluation involving 30 participants further confirms that FIACSO outperforms existing methods in overall aesthetics, compositional harmony, and content integrity. Beyond methodological contributions, this study also offers practical value: it supports photographers in refining image composition without losing context, assists designers in creating balanced layouts with minimal distortion, and provides computational tools to enhance the efficiency and quality of visual media production.

## 1. Introduction

Image aesthetic evaluation and enhancement play a crucial role in advancing the field of visual computing, as they directly influence the quality of image interpretation and its emotional impact on viewers. The ability to assess and optimize the aesthetic elements of an image is vital in various domains, including photography, design, and computer vision, contributing to the creation of visually appealing and contextually meaningful images. Drawing upon aesthetic principles, image optimization encompasses a range of techniques, including image refocusing, brightness/contrast adjustments, and image composition optimization. Among these, image composition stands out as a critical element, reflecting the rational arrangement of elements within the frame and often serving as the primary focal point for appreciating and assessing the aesthetic quality of an image.

The primary aim of composition optimization is to augment the thematic impact of images, specifically aligning with the three fundamental principles of image aesthetics delineated by Luo et al. [1]. Recent researchers have been focusing on aesthetic principles to measure image quality and have increasingly recognized the impact of aesthetics on the overall perception of images, and a series of valuable studies have emerged to fill the void in this element of image optimization [2,3,4]. For instance, Patnaik et al. [5] proposed AesthetiQ, enhancing graphic layouts via multi-modal LLMs’ aesthetic preference alignment, with layout-quality filtering and a new metric. Alsmirat et al. [6] proposed a supervised deep learning-based method for the ideal identification of image retargeting techniques, which utilizes transfer learning to construct deep learning models such as Resnet18, DenseNet121, and InceptionV3 to predict the suitable retargeting method for an input image with a specific resolution. Hong et al. [7] proposed GenCrop a weakly supervised approach to learn high-quality subject-aware cropping from professional stock images by combining them with a pretrained text-to-image diffusion model to generate cropped-uncropped training pairs automatically. Shen et al. [8] proposed a content-aware image retargeting method called PruneRepaint, which incorporates semantic importance for each pixel and an adaptive repainting module to maintain key semantics and achieve local smoothness, outperforming previous approaches in preserving semantics and aesthetics on the RetargetMe benchmark. Hong et al. [9] proposed GenCrop, a weakly supervised subject-aware cropping approach using stock images and a diffusion model to generate training pairs, performing well against supervised and weakly supervised methods. Additionally, studies by Taichi Hussain et al. [10], Hui Wang [11] and Gao et al. [12] have also explored different perspectives and methods to promote the development of image composition optimization.

Presently, these methods can be broadly categorized into three types. The first type is rule-based optimization methods, which focus on applying fundamental composition principles to optimize image composition. Despite their adherence to aesthetic rules, these techniques tend to be complex and may necessitate manual intervention, reducing their efficiency and applicability in automated scenarios. The second type is learning-based optimization methods, which typically involve modeling compositional features using big data and applying these features to generate visually more pleasing images. Unfortunately, such methods mostly rely on cropping to process images, hence they suffer from the drawback of content loss, which may lead to the disappearance of important image details. The third type is example-based optimization methods, which adjust input images based on the composition of reference images. These methods usually require retrieving reference images from a database, but finding images with compositions similar to those of the input image poses a challenging problem, limiting their practicality. Beyond the limitations of these three types of methods, existing image optimization approaches also encounter common practical challenges including compression-induced distortion, imprecise object extraction, and disproportionate proportions caused by cropping, all of which hinder the further improvement of image aesthetic quality.

To address these issues, an IACS method was proposed. The proposed method achieved a balance between composition aesthetics and image resemblance through a unified function, while allowing for precise control via parameter adjustments. In evaluating the composition aesthetic, emphasis was placed on the distance between the main semantic line or salient object and the nearest rule-of-thirds line or central line. For images with prominent semantic lines, a modified Hough transform with length filtering and pixel spacing constraints was employed to detect the main semantic line. Similarly, for images containing salient objects, a salient object detection method based on LCSF was utilized to determine the salient object region. In evaluating the similarity to original images, edge similarity measured by Canny operator was combined with the SSIM for measurement. Furthermore, a framework of image aesthetic evaluation with composition and similarity-based optimization (FIACSO) was proposed. After categorizing the composition of the image, the framework utilized a semantic segmentation network for image segmentation to obtain composition information. Subsequently, the IACS method was applied to optimize and adjust the image. Finally, the optimized segmentation result was refined using DeepSIM, a GAN-based image generation model. The purpose of this step is to ensure that the adjusted composition is seamlessly integrated into the original image, avoiding visual artifacts, boundary discontinuities, or distortions that may occur when only the segmentation result is used. In this way, the GAN produces a more natural and visually consistent final image. In brief, the main contributions of this study are as follows:

1. An image aesthetic evaluation with composition and similarity (IACS) method was proposed. The proposed method achieved a balance between composition aesthetics and image similarity through a unified function while allowing for precise control via parameter adjustments. In composition aesthetic evaluation, emphasis was placed on the distance between the main semantic line or salient object and the nearest rule-of-thirds line or central line. For images with prominent semantic lines, a modified Hough transform with length filtering and pixel spacing constraints was employed to detect the main semantic line. For images containing salient objects, a detection method based on LCSF was employed to determine the salient object region. In evaluating the similarity to original images, edge similarity measured by Canny operator was combined with the SSIM for measurement.
2. A framework of image aesthetic evaluation with composition and similarity-based optimization (FIACSO) was proposed. This framework categorized the composition of the image initially and then utilized a semantic segmentation network to segment the image, extracting composition information. Subsequently, the IACS method was applied to optimize adjustments to the image. Finally, a generative adversarial network (GAN) was employed to generate optimized images that adhered to composition rules and closely resembled the original image.
3. A salient object detection method based on LCSF was introduced. Initially, the image is processed through Gaussian filtering and converted to the Lab color space. Afterwards, threshold segmentation and morphological operations were performed on the brightness channel to calculate saliency features and extract the maximum feature. Then, the GrabCut algorithm was applied for image segmentation to extract the foreground. The resulting foreground was multiplied by the saliency feature to generate a saliency map, effectively highlighting the salient object region within the image.

## 2. Materials and Methods

### 2.1. Main Ideas

In various themes and scenarios, diverse composition techniques are commonly employed. For instance, landscape photography frequently employs linear or symmetrical composition, while portrait photography leans towards central composition or rule of thirds (RoT). This paper delves into optimizing two distinct types of images: those with main semantic lines and those featuring salient objects. These categories of images are pervasive in practical applications and are pivotal in augmenting the visual allure and aesthetic appeal of images.

**Figure 1:**
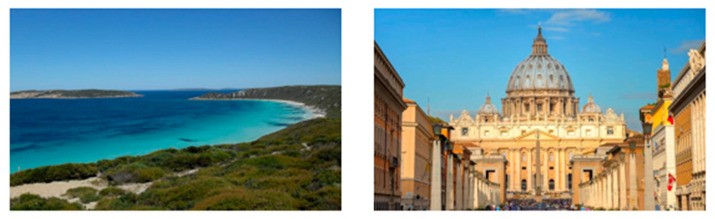
Example of images with semantic lines.

For images featuring main semantic lines, the main semantic line plays a crucial role in directing visual attention and emphasizing the structure of the image. As depicted in Figure 1, when optimizing the composition of such images, the role of semantic lines can be further emphasized, enabling viewers to focus more on the theme and understand the content of the image.

**Figure 2:**
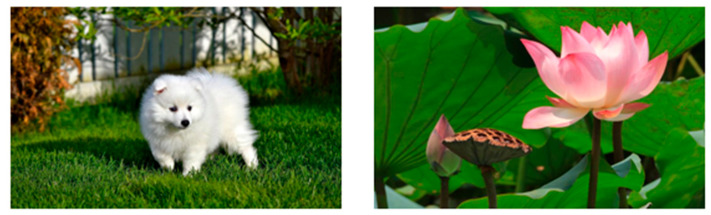
Example of images with salient objects.

For images containing salient objects, the salient object serves as the central element of composition, capturing viewers’ attention at its core. As depicted in Figure 2, optimizing the composition of such images aids in accentuating the salient object, granting it a more prominent presence within the frame.

**Figure 3:**
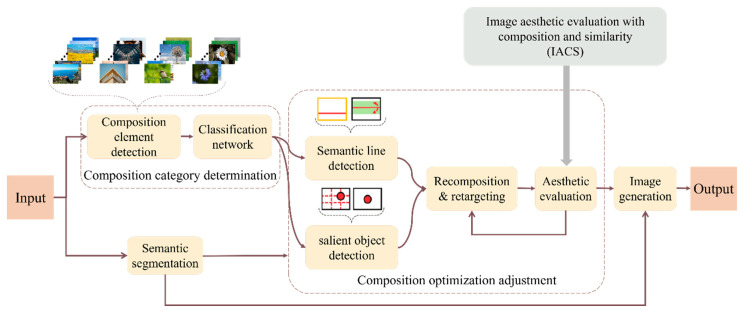
Framework of image aesthetic evaluation with composition and similarity-based optimization (FIACSO) (arrows indicate the flow of the optimization process).

The process of FIACSO for predicted aesthetic score improvement, which integrates composition and similarity, was proposed in this paper and is illustrated in Figure 3.

From Figure 3, it is evident that FIACSO primarily comprises the following components.

1. Composition category determination: In this section, a composition category determination network was designed to detect the composition category of the input image. This network was capable of flexibly identifying complex composition characteristics during the composition category prediction phase and provided confidence scores for adherence to composition rules, thereby demonstrating high accuracy and generalization capabilities.
2. Image aesthetic evaluation with composition and similarity (IACS): In this section, a unified function was used to balance composition aesthetics and image similarity. The evaluation of composition aesthetics focused on the distance between the main semantic line or salient object and the nearest rule-of-thirds line or central line. For images with prominent semantic lines, an modified Hough transform was employed to detect the main semantic line. For images containing salient objects, a salient object detection method based on LCSF was utilized to determine the salient object region. In evaluating the similarity to original images, edge similarity measured by Canny operator was combined with the SSIM for measurement.
3. Composition optimization adjustment: This section focuses on maximizing the aesthetic evaluation of composition while preserving the original semantic and structural information of the image. It involves a series of steps, including content-aware rotation, determining the position of the main semantic line or salient object, and gradually adjusting this position using the IACS method. The ultimate goal is to achieve the highest possible composition aesthetic evaluation.

The main objective of this study is to investigate methods for optimizing image composition. Therefore, special emphasis is placed on three key elements: determining the categories of composition, employing the IACS method for aesthetic evaluation, and making adjustments for composition optimization. Semantic segmentation and image generation methods are not the primary focus of this study. Instead, well-established methods are adopted for both tasks in subsequent experiments. The Swin-Base [13] was utilized as the semantic segmentation network model, while the DeepSIM [14], a generative adversarial network trained on single images, was employed for image generation.

### 2.2. Problem Definition

Unless otherwise specified in the Rules, the relevant definitions are as follows:

$X_{i}$: Original input image.

$Y_{r}$: Optimized output image.

C: Composition category.

$A_{c}$: Composition aesthetic evaluation.

$A_{s}$: Similarity evaluation.

A: Comprehensive evaluation.

$I_{i}$: Semantic segmentation result of image.

$I_{o}$: Intermediate image during the optimization process.

$\lambda_{1},\;\lambda_{2},\;\lambda_{3}$: Weight parameter, with a value range of [0, 1].

L: Length of the semantic line.

L_min: Minimum length threshold of the semantic line.

d: Pixel spacing between points on the semantic line.

d_max: Maximum pixel spacing threshold.

$C=\left\{L_{1},\;L_{2},\;\ldots ,\;L_{n}\right\}$: The collection of candidate main semantic lines.

$L_{i}:$ The main semantic line.

$L^{l}$: The line of thirds or center line closest to line $L_{i}$.

$R_{i}$: The region of the salient object.

$p_{i}$: The center point of $R_{i}$.

$p_{i}^{s}$: The intersection point closest to the line of thirds or the center line, nearest to point $p_{i}$.

$D_{i}$: The central axis of $R_{i}$.

$D^{l}$: The line of thirds or center line closest to line $D_{i}$.

dis: Euclidean distance.

l, c, s: Luminance, contrast, and structure.

$\mu_{I_{i}}$: The mean of $I_{i}$.

$\mu_{I_{o}}$: The mean of $I_{o}$.

$\sigma_{I_{i}}$: The standard deviation of $I_{i}$.

$\sigma_{I_{o}}$: The standard deviation of $I_{o}$.

$\sigma_{I_{o}I_{i}}$: The covariance between $I_{o}$ and $I_{i}$.

$I_{o}^{\prime }$: The edge detection results of the image during the optimization process.

$I_{i}^{\prime }$: The edge detection results of the semantic segmentation result image.

$\sigma_{I_{o}^{\prime }}$: The standard deviation of $I_{o}^{\prime }$.

$\sigma_{I_{i}^{\prime }}$: The standard deviation of $I_{i}^{\prime }$.

$\sigma_{I_{o}^{\prime }I_{i}^{\prime }}$: The covariance between $I_{o}^{\prime }$ and $I_{i}^{\prime }$.

$C_{1}$, $C_{2}$, $C_{3},C_{3}^{\prime }$: Ensure the stability of calculations by incorporating constants to prevent instability when the denominator approaches zero.

$A_{max}$: The initial IACS score.

X: The initial number of datasets.

$X=\left\{X,\;T_{\theta }X\right\}$: The number of datasets after data augmentation.

The original image $X_{i}$ is initially categorized into composition category C, followed by semantic segmentation to obtain the segmented result image $I_{i}$ containing composition information. Subsequently, the image composition is incrementally adjusted using the IACS method, resulting in the adjusted image $I_{o}$. Finally, a generative adversarial network combines the original image $X_{i}$ with the adjusted image $I_{o}$ to generate the image, producing the optimized result image $Y_{r}$. This process enhances the composition aesthetics while preserving the content and features of the original image.

### 2.3. Composition Category Determination

**Figure 4:**
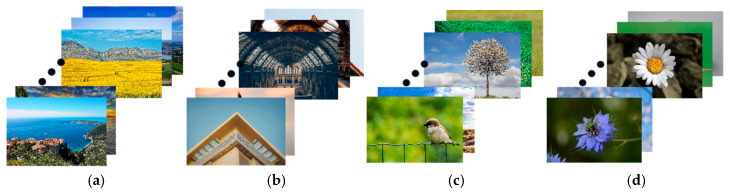
Four composition categories: (**a**) Linear, (**b**) Symmetric, (**c**) RoT, (**d**) Center.

In the domain of image composition optimization, prior research has predominantly focused on RoT and central composition methods [15]. However, this approach is relatively narrow and fails to encompass the full diversity and richness of image composition. To address this limitation, the current study embarks on a more extensive exploration, identifying linear, symmetrical, RoT, and central compositions as the primary categories. These four types of compositions integrate a wide range of common compositional techniques and principles, providing a comprehensive framework for design. Consequently, this study extends beyond previous studies by incorporating a broader array of factors in composition optimization, delivering deeper and more comprehensive insights into the art of image composition. A schematic illustration is provided in Figure 4.

**Figure 5:**
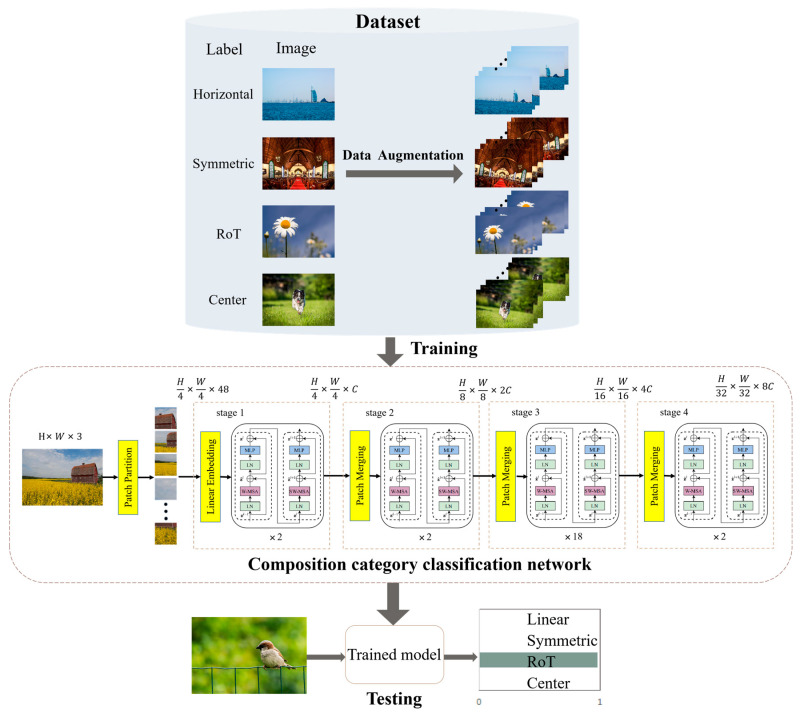
Flowchart for determining compositional categories.

To select an appropriate composition optimization module, it is essential to employ a network capable of determining the composition category for input images. The approach to choosing the composition categorization model in this study includes several key elements:

1. Image labeling and enhancement: This study categorized and labeled images from the composition classification dataset into four types: linear, symmetrical, RoT, and central compositions. All images were front-facing (i.e., horizontally oriented) professional photos without any skew. However, images captured in everyday situations often did not adhere to composition norms or horizontal alignment principles. Therefore, this paper introduced a data augmentation step involving random rotations of ±10 degrees to enhance the model’s ability to generalize [16].
2. Model selection and tuning: To identify the optimal network model for recognizing composition rules, this study experimentally evaluated various well-known CNN and transformer-based models. After fine-tuning, Swin-Base was selected as the most suitable model. Further details are available in Figure 5 of the experimental section.
3. Model Training: The AdamW optimizer [17] was utilized during the training process. Hyperparameters, learning rate ranges, and convergence strategies were meticulously set to promote efficient learning of composition rules. Recognizing that an image might conform to multiple composition rules, a multi-label handling strategy was implemented: in each training epoch, one label was selected as the ground truth. During testing, a prediction was deemed correct if it corresponded to any of the image’s applicable labels. The model predicted by issuing confidence scores for each of the four composition categories. By establishing thresholds and decision criteria, the model adeptly recognized and managed images with complex composition features, thereby enhancing the accuracy and robustness of the composition categorization.

### 2.4. Image Aesthetic Evaluation with Composition and Similarity IACS

To ensure the quality of output images, optimization focused on two essential attributes. Firstly, the image composition was optimized according to specific compositional rules. Secondly, the optimized image preserved as much information from the original image as possible while minimizing visual flaws or distortions. To address these requirements, an IACS method was proposed. This method combined the aesthetics of image composition and the similarity between the final and the original images into a unified function, calculated according to the following Equation (1):

(1) $$A\left(I_{o}\right)=\lambda_{1}\cdot A_{c}\left(I_{o}\right)+\left(1-\lambda_{1}\right)\cdot A_{s}\left(I_{o},I_{i}\right)$$

where $A_{c}$ represents the evaluation of the compositional aesthetics of the optimized output image $I_{o}$. $A_{s}$ represents the similarity assessment between $I_{o}$ and the input image $I_{i}$. The parameter $\lambda_{1}\in$ [0, 1] regulates the impact of these two elements. The objective is to maximize the value of $I_{o}$. A higher $\lambda_{1}$ value enhances the compositional quality of the output, while a lower $\lambda_{1}$ maintains closer similarity to the input image. $\lambda_{1}$ was determined as 0.5 through the sensitivity analysis presented in Section 3.4.1. This value maximizes both the IACS comprehensive score (*A* = 0.80 ± 0.04) and subjective preference rate (82 ± 4%), achieving an optimal balance between compositional aesthetic enhancement and original content preservation.

#### 2.4.1. Composition Aesthetic Evaluation Based on Main Semantic Line

Linear and Symmetric compositions are particularly suited for images with semantic lines, including landscapes or architectural scenes. A direct and effective method to evaluate such compositions is by detecting the main semantic line and assessing its aesthetic quality using $A_{c}\left(I\right)$.

Since the results of semantic segmentation may include multiple semantic lines, it is necessary to identify one main semantic line based on its position for aesthetic evaluation.

The Hough Transform [18] can detect semantic lines, including short and discontinuous ones. The core of the Hough Transform for line detection relies on the Hough theorem, which maps line detection in the image space (x, y) to peak detection in the parameter space (ρ, θ). The mathematical expression of the Hough theorem for line detection is given in Equation (2):

(2) ρ=xcosθ+ysinθ

where ρ denotes the perpendicular distance from the origin of the image coordinate system to the straight line, and θ denotes the angle between the perpendicular line and the *x*-axis. In Hough space, each pixel (x, y) in the image space corresponds to a sinusoidal curve; the intersection of multiple sinusoidal curves in Hough space indicates that these pixels belong to the same straight line in the image space.

However, the standard Hough Transform may detect short or discontinuous lines that are not suitable as main semantic lines. Thus, this paper modified the Hough Transform as follows:

1. Semantic Line Length Filtering: To exclude overly short semantic lines, a minimum length threshold L_min was established. Any detected semantic line, represented by length L, must meet the criteria of Equation (3) to be considered:
  (3) L≥L_min
2. Pixel Spacing Threshold: To ensure continuity of semantic lines, a maximum pixel spacing threshold d_max was established. For a segment formed by pixels $\left(x_{1},\;y_{1}\right)$ and $\left(x_{2},\;y_{2}\right)$, their distance must satisfy Equation (4):
  (4) $$\sqrt{{\left(x_{2}-x_{1}\right)}^{2}+{\left(y_{2}-y_{1}\right)}^{2}}\leq d_{max}$$

Determining the Main Semantic Line: Based on the filtering conditions from the first two steps, a semantic line $L_{i}$ that meets both conditions is selected as the main semantic line from the candidate set $C\;=\;\left\{L_{1},\;L_{2},\;\ldots ,\;L_{n}\right\}$.

In evaluating composition aesthetics based on the main semantic line, the image was first converted to grayscale and processed through Canny edge detection [19] to produce a binary image. The modified Hough Transform was then used to detect the main semantic line. Finally, the compositional aesthetic evaluation is calculated by utilizing the Euclidean distance between the main semantic line and the nearest rule-of-thirds line or central line.

For linear composition, the alignment of the main semantic line with both horizontal and vertical rule-of-thirds lines is evaluated, and the axis yielding the smaller normalized distance is selected. The corresponding compositional aesthetic score $A_{c}$ is given by Equation (5):

(5) $$A_{c}\left(I\right)=\cos\left(2\cdot \min\left\{\frac{dis\left(L_{i},\;nearest\left(\left\{x=w/3,2w/3\right\}\right)\right)}{w/3},\frac{dis\left(L_{i},\;nearest\left(\left\{y=h/3,2h/3\right\}\right)\right)}{h/3}\right\}\cdot \frac{\pi }{2}\right)$$

where w and h respectively represent the width and height of the image, $L_{i}$ is the main semantic line, dis(·, ·) is the perpendicular Euclidean distance between lines.

For symmetrical composition, the alignment of the main semantic line with both the vertical and horizontal central lines is evaluated, and the corresponding compositional aesthetic score $A_{c}$ is given by Equation (6):

(6) $$A_{c}\left(I\right)=\cos\left(2\cdot \min\left\{\frac{dis\left(L_{i},x=w/2\right)}{w/2},\frac{dis\left(L_{i},y=h/2\right)}{h/2}\right\}\cdot \frac{\pi }{2}\right)$$

#### 2.4.2. Composition Aesthetic Evaluation Based on Salient Object

RoT and center Composition are widely used in image composition for photos with clear foreground salient objects. Such images are particularly popular in personal photo collections, including pictures of family members, friends, pets, and interesting objects like flowers.

Detecting the salient object region is crucial for this type of composition and subsequent optimization. To this end, a salient object detection method based on LCSF was proposed to accurately distinguish between the foreground and background of an image, aiming to highlight the salient object region. The method involved processing the semantic segmentation results using a Gaussian filter and converting the image to the Lab color space. The luminance channel was then subjected to threshold segmentation and morphological operations to calculate salience features and extract the maximum features.

The calculation of salience features based on the luminance channel is given by Equation (7):

(7) $$I_{salient\;}\left(x,y\right)=\max\left(0,L\left(x,y\right)-\frac{1}{k}\underset{\left(i,\;j\right)\in \Omega \left(x,\;y\right)}{\sum }L\left(i,j\right)\right)$$

where $I_{salient\;}\left(x,\;y\right)$ represents the salience feature value at pixel (x, y); L(x, y) represents the luminance value of pixel (x, y) in the Lab color space; Ω(*x*, *y*) represents a 3 × 3 local neighborhood centered at (x, y); K = 9 represents the total number of pixels in the neighborhood; max(0, ·) is used to suppress negative differences and retain pixels with higher luminance than the local average.

Subsequently, the GrabCut algorithm [20] was used to segment the image, extract the foreground, and multiply it by the salience features. The fusion process of the foreground region and salience features is given by Equation (8):

(8) $$I_{salient\_image}\left(x,\;y\right)=I_{foreground}\left(x,\;y\right)\cdot I_{salient}\left(x,\;y\right)$$

where $I_{foreground}\left(x,\;y\right)$ represents the foreground mask output by GrabCut; $I_{salient\_image}\left(x,\;y\right)$ represents the final salient object image. This formula enhances salient features in the foreground region through pixel-wise multiplication while suppressing background noise, effectively enhancing the visibility of the salient object region in the image.

The process of salient object region detection method based on LCSF proposed in this study is illustrated in Algorithm 1.
**Algorithm 1.** Salient object region detection based on LCSFInput: I
Output: $I_{\mathrm{salient}\_\mathrm{object}\_\mathrm{image}\;}$
function SalientObjectDetection (I)
${\;\;\;I}_{\mathrm{smooth}}\leftarrow \mathrm{GaussianFilter}\;\left(I,\;G\right)$$\;\;\;L,a,b\leftarrow \;\left(I_{\mathrm{smooth}}\right)$${\;\;\;I}_{\mathrm{brightness}}\leftarrow L$${\;\;\;I}_{\mathrm{binary}}\leftarrow {\mathrm{Thresholding}\;(I}_{\mathrm{brightness}},T)$${\;\;\;I}_{\mathrm{closed}}\leftarrow {\mathrm{Closing}\;(I}_{\mathrm{binary}})$${\;\;\;I}_{\mathrm{salient}}\leftarrow {\mathrm{ComputeSalientFeature}\;(I}_{\mathrm{closed}})$${\;\;\;I}_{\mathrm{mask}}\leftarrow {\mathrm{CreateMask}\;(I,\;I}_{\mathrm{salient}})$${\;\;\;I}_{\mathrm{foreground}}\leftarrow \mathrm{GrabCut}(I,I_{\mathrm{mask}})$${\;\;\;I}_{\mathrm{salient}\_\mathrm{image}}\leftarrow I_{\mathrm{foreground}}{\;\times \;I}_{\mathrm{salient}}$return $I_{salient\_object\_image}$
end functionMain:I←Input Image$I_{\mathrm{salient}\_\mathrm{object}\_\mathrm{image}}\leftarrow \mathrm{SalientObjectDetection}\left(I\right)$

Based on the detected salient object region, two elements need to be considered when calculating compositional aesthetics. The first element is the distance from the salient object to the four points of intersection of the rule-of-thirds or the center point in the image. The second element is whether the salient object is placed along the lines of the rule-of-thirds or the center line. For an image, the calculation was done according to the following Equation (9):

(9) $$A_{c}\left(I\right)=\lambda_{2}\cdot A_{dis}\left(I\right)+\left(1-\lambda_{2}\right)\cdot A_{pos}\left(I\right)$$

where $A_{dis}$ and $A_{pos}$ respectively consider the aforementioned elements. The parameter $\lambda_{2}$∈ [0, 1] controls the influence of $A_{dis}$ and $A_{pos}$. Through the sensitivity analysis presented in Section 3.4.2, α = 1/3 was identified as optimal: it prioritizes $A_{pos}$ while retaining a moderate weight for $A_{dis}$, resulting in the highest average $A_{c}$ (0.83 ± 0.07) and subjective aesthetic score (4.2 ± 0.2).

For the rule-of-thirds composition, its parameter $A_{dis}$ is given by Equation (10):

(10) $$A_{dis}\left(I\right)=cos\left(\left(\frac{\left|p_{ix}-p_{ix}^{s}\right|}{w/3}+\frac{\left|p_{iy}-p_{iy}^{s}\right|}{h/3}\right)\cdot \frac{\pi }{2}\right)$$

where $p_{i}$ and $p_{i}^{s}$ respectively represent the center point of the salient object region $R_{i}$ and the intersection point closest to $p_{i}$.

For central composition, its similarity evaluation score $A_{dis}$ is given by Equation (11):

(11) $$A_{dis}\left(I\right)=cos\left(\left(\frac{\left|p_{ix}-p_{ix}^{s}\right|}{w/2}+\frac{\left|p_{iy}-p_{iy}^{s}\right|}{h/2}\right)\cdot \frac{\pi }{2}\right)$$

According to the above formulas, when the center of the main object in the image aligns with one of the intersection points, the value of $A_{dis}$ is 1.

For most images with prominent salient objects, including a person or a tall building, the central axis is nearly vertical. Therefore, this paper calculates the central axis by dividing the salient object into two equal regions using a vertical line.

For rule-of-thirds composition, its similarity evaluation score $A_{pos}$ is given by Equation (12):

(12) $$A_{pos}\left(I\right)=\cos\left(2\cdot \min\left\{\frac{dis\left(D_{i},\;nearest\left(\left\{x=w/3,2w/3\right\}\right)\right)}{w/3},\frac{dis\left(D_{i},\;nearest\left(\left\{y=h/3,2h/3\right\}\right)\right)}{h/3}\right\}\cdot \frac{\pi }{2}\right)$$

where $D_{i}$ represents the central axis of the salient object region $R_{i}$.

For central composition, its similarity evaluation score $A_{pos}$ is given by Equation (13):

(13) $$A_{pos}\left(I\right)=\cos\left(2\cdot \min\left\{\frac{dis\left(D_{i},x=w/2\right)}{w/2},\frac{dis\left(D_{i},y=h/2\right)}{h/2}\right\}\cdot \frac{\pi }{2}\right)$$

where $D_{i}$ represent the central axis of the salient object region $R_{i}$.

#### 2.4.3. Evaluation of Similarity to Original Images

Image retargeting is a technique used to adjust the position of semantic lines or foreground salient objects in order to enhance compositional aesthetics. During this process, visual distortion affects the image, particularly when dealing with images featuring complex background structures. To manage this distortion within an acceptable range, this paper employed similarity measurement to quantify the visual variances between the optimized and original images.

While traditional quality metrics like mean square error (MSE) are computationally simple, they fail to accurately reflect visual quality from the perceptual standpoint. Hence, in some studies, SSIM [21] has been widely used to assess perceptual image similarity. Unlike MSE, SSIM is a perceptual model that aligns more closely with human visual perception. SSIM is calculated according to the following Equation (14):

(14) $$SSIM\left(I_{o},I_{i}\right)={\left[l\left(I_{o},\;I_{i}\right)\right]}^{\alpha }\cdot {\left[c\left(I_{o},\;I_{i}\right)\right]}^{\beta }\cdot {\left[s\left(I_{o},\;I_{i}\right)\right]}^{\gamma }$$

where l, c and s respectively represent the luminance, contrast, and structure between $I_{o}$ and $I_{i}$.

The SSIM value typically ranges from 0 to 1, with values closer to 1 indicating greater similarity between the two images. The individual modules are calculated using the following Equation (15):

(15) $$\;\;\begin{array}{l} l\left(I_{o},\;I_{i}\right)=\frac{2\mu_{I_{o}}\mu_{I_{i}}+C_{1}}{\mu_{I_{o}}^{2}+\mu_{I_{i}}^{2}+C_{1}}\\ c\left(I_{o},\;I_{i}\right)=\frac{2\sigma_{I_{o}}\sigma_{I_{i}}+C_{2}}{\sigma_{I_{o}}^{2}+\sigma_{I_{i}}^{2}+C_{2}}\\ s\left(I_{o},\;I_{i}\right)=\frac{\sigma_{I_{o}I_{i}}+C_{3}}{\sigma_{I_{o}}\sigma_{I_{i}}+C_{3}} \end{array}$$

where $\mu_{I_{o}}$ and where $\mu_{I_{i}}$ respectively represent the means of $\sigma_{I_{o}}$ and $\sigma_{I_{i}}$, while $\sigma_{I_{o}}$ and $\sigma_{I_{i}}$ represent their respective standard deviations. $\sigma_{I_{o}I_{i}}$ represents the covariance between $I_{o}$ and $I_{i}$. $C_{1}$, $C_{2}$ and $C_{3}$ are constants used to ensure computational stability and prevent instability when the denominator approaches zero.

However, the evaluation of edge structures lacks correlation in the structural section of SSIM, making it difficult to measure differences in edge and contour structure information between images [22]. Because the human visual system is most sensitive to edge and contour structure information, this paper improves upon SSIM by adding a measure of edge similarity. Canny edge detection was applied to both the semantic segmentation result image and the image obtained during the optimization process to generate edge maps. Subsequently, we compute the edge similarity based on Equation (16):

(16) $$e\left(I_{o},\;I_{i}\right)=\frac{\sigma_{I_{o}^{\prime }I_{i}^{\prime }}+C_{3}^{\prime }}{\sigma_{I_{o}^{\prime }}\sigma_{I_{i}^{\prime }}+C_{3}^{\prime }}$$

where $\sigma_{I_{o}^{\prime }}$ and $\sigma_{I_{i}^{\prime }}$ respectively represent the standard deviation of the edge detection result images $I_{o}^{\prime }$ and $I_{i}^{\prime }$, and $\sigma_{I_{o}^{\prime }}\sigma_{I_{i}^{\prime }}$ represents their covariance. The constant $C_{3}^{\prime }$ is a constant that ensures computational stability.

By combining Equations (14)–(16), the equation for calculating the similarity between the images $A_{S}\left(I_{o},\;I_{i}\right)$ was obtained as shown in Equation (17):

(17) $$A_{S}\left(I_{o},\;I_{i}\right)=l\left(I_{o},\;I_{i}\right)\cdot c\left(I_{o},\;I_{i}\right)\cdot \left(\lambda_{3}\cdot s\left(I_{o},\;I_{i}\right)+\left(1-\lambda_{3}\right)\cdot e\left(I_{o},\;I_{i}\right)\right)\;={\left[\frac{2\mu_{I_{o}}\mu_{I_{i}}+C_{1}}{\mu_{I_{o}}^{2}+\mu_{I_{i}}^{2}+C_{1}}\right]}^{\alpha }\cdot {\left[\frac{2\sigma_{I_{o}}\sigma_{I_{i}}+C_{2}}{\sigma_{I_{o}}^{2}+\sigma_{I_{i}}^{2}+C_{2}}\right]}^{\beta }\cdot {\left[\lambda_{3}\cdot \left[\frac{\sigma_{I_{o}I_{i}}+C_{3}}{\sigma_{I_{o}}\sigma_{I_{i}}+C_{3}}\right]+\left(1-\lambda_{3}\right)\cdot \left[\frac{\;\sigma_{I_{o}^{\prime }I_{i}^{\prime }}+C_{3}^{\prime }}{\sigma_{I_{o}^{\prime }}\sigma_{I_{i}^{\prime }}+C_{3}^{\prime }}\right]\right]}^{\gamma }$$

where α, β and γ were set to 1 to control the relative importance of the three components. The parameter $\lambda_{3}\;\in$ [0, 1] governs the influence of image structure and edges, and its value is set to 0.5 to balance the two factors. Typically, the constants are defined as $C_{1}\;=\;{\left(K_{1}L\right)}^{2}$, $C_{2}\;=\;{\left(K_{2}L\right)}^{2}$, and $C_{3}\;=\;C_{2}/2$, where $K_{1}\;=\;0.01$, $K_{2\;}=\;0.03$, and L = 255. In this experiment, $C_{3}^{\prime }\;=\;$1 × 10^−4^ was introduced to ensure numerical stability.

### 2.5. Composition Optimization Adjustment

The optimized image is the one that maximizes the function $A\left(I_{o}\right)$ as calculated by Equation (18):

(18) $$A\left(I_{o}\right)=arg\underset{I_{o}}{max}A\left(I_{o}\right)$$

The solution is found through the following steps:

1. Image rotation. Any angle of tilt can disrupt the balance in the image. After detecting the main semantic lines, the image needs to be adjusted to the correct orientation. Rotation can result in the loss of some semantic information. To address this, a content-aware rotation [23] method was adopted, which maintains the element ratio and preserves the semantic and structural information of the original image to the greatest extent. This step is only applied to images with linear or symmetrical compositions.
2. Determine the optimal position $P_{c}$ of the main semantic line or salient object for compositional aesthetic evaluation $A_{c}\left(I\right)$. Based on the semantic image $I_{i}$ of the original image, identify the main semantic line or salient object, and use the image composition aesthetic evaluation equation $A_{c}\left(I\right)$ to maximize this function to find the optimal position of the main semantic line or salient object $P_{c}$, disregarding similarity to the original image. Practically, the optimal position is the closest line of thirds or center line to the main semantic line or salient object of the image.
3. Determine the adjustment position of the main semantic line or salient object for this round. In the semantic image $I_{i}$ of the original image, adjust the main semantic line or salient object from its original position $P_{s}$, which is the position with the highest similarity, to the optimal position $P_{c}$ determined in step (2).
4. Adjust the main semantic line or salient object in the semantic image. The Seam Carving method [24] is employed in this paper, selectively removing or inserting rows of pixels (Seams) without altering the image size while preserving important content, to move the position of the main semantic line or salient object, resulting in the adjusted semantic image $I_{o}$. Compared to traditional image cropping and scaling techniques, Seam Carving more effectively preserves image information without reducing image resolution.
5. Compositional aesthetic evaluation based on IACS. Calculate the aesthetics score A(I) for the semantic image after adjusting the position of the main semantic line or salient object using the IACS aesthetic evaluation method.
6. If the adjustment position of the main semantic line or salient object has not reached the optimal position $A_{c}\left(I\right)$ determined in step (2), return to step (3); otherwise, proceed to step (7).
7. Among all the semantic images adjusted during the process, the one with the highest IACS evaluation A(I) is the final result of the composition optimization adjustment, with the position of the semantic line or salient object marked as $P_{o}$.

During the adjustment process, the aesthetic evaluation result $A_{c}\left(I\right)$ continuously improves, while the similarity score to the original image $A_{s}\left(I\right)$ gradually decreases. The position where the combined IACS aesthetic evaluation A(I) reaches its maximum is the optimal position for compositional optimization adjustment of the main semantic line or salient object in the input image. At this point, the image was optimized according to specific compositional rules while retaining as much of the original image’s information as possible.

### 2.6. The Process of FIACSO

By following the steps outlined above, the process of the framework of image aesthetic evaluation with composition and similarity-based optimization (FIACSO) is detailed in Algorithm 2.
**Algorithm 2.** The process of the framework of image aesthetic evaluation with composition and similarity-based optimization (FIACSO)Input: $X_{i}$
Output: $Y_{r}$
function ${\mathrm{CompositionClassification}\;(X}_{i})$${\;\;\;\mathrm{Classify}\;\mathrm{the}\;\mathrm{composition}\;\mathrm{category}\;\mathrm{of}\;\mathrm{input}\;\mathrm{image}\;X}_{i}$   Obtain composition category label Creturn C$\mathrm{function}\;{\mathrm{SemanticSegmentation}\;(X}_{i})$${\;\;\;\mathrm{Perform}\;\mathrm{semantic}\;\mathrm{segmentation}\;\mathrm{on}\;\mathrm{input}\;\mathrm{image}\;X}_{i}$${\;\;\;\mathrm{Obtain}\;\mathrm{segmented}\;\mathrm{result}\;\mathrm{image}\;I}_{i}$${\mathrm{return}\;I}_{i}$${\mathrm{function}\;\mathrm{CompositionOptimizationAdjustment}\;(I}_{i},\;C)$${\;\;\;I}_{o}\leftarrow I_{i}$$\;\;\;\lambda_{1}\in \;[0,\;1]▹\;\mathrm{Control}\;\mathrm{the}\;\mathrm{influence}\;\mathrm{of}\;\mathrm{composition}\;\mathrm{aesthetics}\;\mathrm{and}\;\mathrm{image}\;\mathrm{similarity}$$\;\;\;A_{\max}\leftarrow -\infty$   improvement ← true    while improvement do${\;\;\;\;\;\;\;\;A}_{c}\leftarrow {\;\mathrm{EvaluateComposition}\;(I}_{o},\;C)$${\;\;\;\;\;\;\;\;A}_{s}\leftarrow {\;\mathrm{EvaluateSimilarity}\;(I}_{o}{,\;I}_{i})$${\;\;\;\;\;\;\;\;A(I}_{o})\leftarrow \lambda_{1}{\;\times \;A}_{c}+(1{-\lambda }_{1}{)\;\times \;A}_{s}$${\;\;\;\;\;\;\;\;\mathrm{if}\;A(I}_{o}{)\;>\;A}_{\max}\;\mathrm{then}$${\;\;\;\;\;\;\;\;\;\;\;\;A}_{\max}\leftarrow {A(I}_{o})$${\;\;\;\;\;\;\;\;\;\;\;\;I}_{o}\leftarrow {\;\mathrm{ApplyOptimizationAdjustment}\;(I}_{o})$            improvement ← true         else            improvement ← false         end if   end while$\;\;\;{\mathrm{return}\;I}_{o}$end function$\mathrm{function}\;{\mathrm{GenerationOfOptimizedImage}\;(I}_{o}{,\;X}_{i})$$\;\;\;\mathrm{Combine}\;\mathrm{adjusted}\;\mathrm{image}\;I_{o}\;\mathrm{with}\;\mathrm{input}\;\mathrm{image}\;X_{i}\;\mathrm{to}\;\mathrm{generate}\;\mathrm{Optimized}\;\mathrm{output}\;\mathrm{image}\;Y_{r}$$\;\;\;\mathrm{return}\;Y_{r}$end functionMain:$C\leftarrow \;\mathrm{CompositionClassification}\;(X_{i})$$I_{i}\leftarrow \;\mathrm{SemanticSegmentation}\;(X_{i})$$I_{o}\leftarrow \;\mathrm{CompositionOptimizationAdjustment}\;(I_{i},\;C)$$Y_{r}\leftarrow {\;\mathrm{GenerationOfOptimizedImage}\;(I}_{o}{,\;X}_{i})$

1. Composition category determination: First, the input image $X_{i}$ undergoes a determination of its composition category C. This step involves analyzing the image’s features and attributes, classifying it into a specific composition type, and obtaining the corresponding composition category label C. This process is implemented by the function CompositionClassification.
2. Semantic segmentation: Next, a trained semantic segmentation network is used to segment the input image $X_{i}$, resulting in a segmented image that contains semantic and compositional information. This process is implemented by the function SemanticSegmentation.
3. Compositional optimization adjustment: In the compositional optimization adjustment stage, the algorithm incrementally adjusts the image’s composition to enhance the aesthetic score while maintaining image similarity. This process is implemented by the function CompositionOptimizationAdustment. Initially, the segmented image $I_{i}$ is used as the initial optimized image $I_{o}$, and a parameter $\lambda_{1}$ is set to control the influence of compositional aesthetics and image similarity. Then, through a cyclic iterative process, evaluate the adjusted image $I_{o}$’s compositional aesthetic score $A_{c}$ and its similarity assessment $A_{s}$ to the image $I_{i}$, the algorithm decides whether to accept the adjusted image and makes compositional adjustments under the premise of preserving the image content, until the optimal compositional effect is achieved.
4. Generation of the optimized image: Finally, by combining the original image $X_{i}$ and the adjusted image $I_{o}$, the final optimized output image $Y_{r}$ is produced. This process is completed by the function GenerationOfOptimizedImage.

The entire algorithm framework considers both compositional aesthetics and image similarity, optimizing the image composition under the premise of maintaining content integrity, effectively enhancing the overall aesthetic quality of the image.

## 3. Experiments

### 3.1. Experimental Software and Hardware Configurations

The training and testing of the network models in this paper were conducted using the PyTorch deep learning framework. The specific experimental hardware and software configuration is shown in Table 1.

### 3.2. Experimental Datasets

Two fundamental datasets, namely the KU_PCP dataset [25] and the ImageNet dataset [26] were utilized in this paper. The KU_PCP dataset was employed to conduct experiments to determine the network’s selection of composition categories. To mitigate overfitting risks associated with training on a small dataset, the network was pretrained on the large-scale ImageNet dataset for image classification. Subsequently, fine-tuning took place on the KU_PCP dataset to refine composition-related elements.

(1) KU_PCP dataset

The KU_PCP dataset consists of images sourced from social sharing platforms and encompasses 4244 landscape images that adhere to diverse composition rules. The composition types are annotated by 18 annotators, with each image being assigned a corresponding label indicating its composition type. For the purpose of the network’s composition category determination, this paper employs images representing four composition methods: Linear, Symmetric, RoT, and Center.

(2) ImageNet dataset

The ImageNet dataset is a comprehensive visual recognition dataset, containing over 14 million images. Each image is associated with a class label encompassing more than 20,000 categories, spanning various domains including animals and transportation. Widely employed in the field of computer vision, particularly in tasks like image classification and salient object detection, this dataset plays a crucial role.

#### Data Preprocessing and Augmentation

To ensure consistency and quality of the input data, all images were first resized to 256 × 256 pixels. When necessary, symmetric padding was applied to preserve the original element ratio, and pixel values were normalized to the range [0, 1] to accelerate convergence and improve training stability. These steps ensured that the input data were standardized and suitable for training deep neural networks.

**Figure 6:**
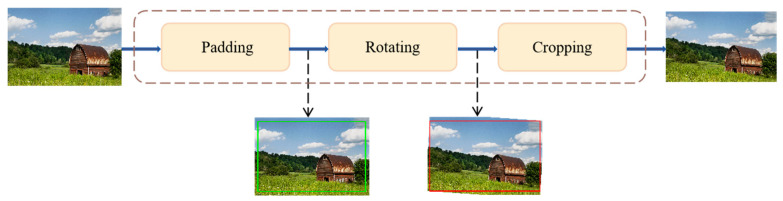
Data augmentation scheme.

In addition to preprocessing, data augmentation was applied to enhance generalization and alleviate the imbalance of composition categories in the KU_PCP dataset. The augmentation strategies included random rotations within ±10 degrees, horizontal flipping, and slight variations in brightness, contrast, and saturation, as illustrated in Figure 6.

These operations effectively expanded the dataset and introduced variations resembling real-world conditions including handheld camera skew or illumination changes. After data augmentation, the scale of the dataset is approximately three times that of the original, and the distribution of the four composition categories becomes more balanced, as presented in Table 2.

### 3.3. Determining the Network’s Selection of Composition Categories

This section evaluates the suitability of the Swin-Base for categorizing image compositions, and it utilizes the enhanced KU_PCP dataset to compare well-known CNN and transformer-based models. The study focuses on various composition types: linear, symmetrical, RoT, and central. CNN models including ResNet [27], ResNext [28], MobileNet [29], and EfficientNet [30]. Transformer models including ViT Transformer [31], Swin Transformer, and MobileViT [32]. Each model was fine-tuned with the final classification layer replaced to adapt to the four composition categories.

To ensure robust training and minimize overfitting, we employed a systematic parameter tuning process. The AdamW optimizer was used with an initial learning rate of 1 × 10^−4^, and a cosine annealing learning rate schedule gradually reduced the learning rate as training progressed. A batch size of 32 was chosen after empirical comparison, providing a good balance between convergence stability and computational efficiency. Early stopping with a patience of 15 epochs was introduced to prevent overfitting, while maintaining sufficient opportunity for convergence. All models were pretrained on ImageNet, and comparative results between CNN and Transformer models are shown in Table 3.

From Table 3, it is evident that Transformer models generally outperform CNN models in terms of cross-validation accuracy, with CNN models averaging 78.96% and Transformer models achieving 80.80%. Notably, the Swin-Base excelled, achieving a cross-validation accuracy of 86.96%. Consequently, Swin-Base proves to be particularly effective for classifying image compositions.

### 3.4. Parameter Sensitivity Analysis

To address potential concerns regarding the arbitrariness in selecting key parameters $\lambda_{1}$ and $\lambda_{2}$, as well as to validate the rationality of these two key parameters, a comprehensive sensitivity analysis was conducted on the augmented KU_PCP dataset. This section elaborates on the experimental design, presents the corresponding analysis results, and systematically clarifies the process for determining the optimal values of two core parameters: $\lambda_{1}$ is responsible for balancing composition aesthetics and image similarity, while $\lambda_{2}$ focuses on balancing distance and position in the context of salient-object-based aesthetic assessment.

#### 3.4.1. Sensitivity of $\lambda_{1}$

The parameter $\lambda_{1}$ ∈ [0, 1] regulates the trade-off between composition aesthetics $A_{c}$ and image similarity $A_{s}$ in the IACS comprehensive evaluation. In the experiment, five representative values of $\lambda_{1}$—0.1, 0.3, 0.5, 0.7, and 0.9—were tested to cover low, medium, and high weights. A total of 100 images were randomly selected from the augmented KU_PCP dataset, with 25 images drawn from each composition category: Linear, Symmetric, RoT, and Center, thereby ensuring consistency with the main experiments. Evaluation metrics comprised objective indicators, including the average values of $A_{c}$, $A_{s}$, and the overall IACS score *A*, as well as subjective validation. For the latter, 10 volunteers, five with photography expertise and five without, assessed the images by providing binary ratings: a score of 1 indicated preference and a score of 0 indicated non-preference, reflecting the perceived balance between aesthetic quality and content.

Table 4 summarizes the results. $\lambda_{1}$ = 0.5 achieved the highest average *A* (0.80 ± 0.04) and subjective preference rate (82% ± 4%): lower $\lambda_{1}$ values (0.1, 0.3) prioritized similarity ($A_{s}$ ≥ 0.85) but failed to enhance aesthetics ($A_{c}$ ≤ 0.63), while higher $\lambda_{1}$ values (0.7, 0.9) maximized $A_{c}$ (≥0.91) but caused severe similarity loss ($A_{s}$ ≤ 0.58) and visual distortion. Thus, $\lambda_{1}$ = 0.5 is confirmed as optimal for balancing aesthetics and similarity.

#### 3.4.2. Sensitivity of $\lambda_{2}$

The parameter $\lambda_{2}$ ∈ [0, 1] balances two components of $A_{c}$ for RoT and Center compositions, where $A_{dis}$ denotes the distance from the salient object’s center to the nearest rule intersection and $A_{pos}$ represents the alignment of the salient object’s central axis with rule lines. To isolate the impact of $A_{dis}$ and $A_{pos}$, five values of $\lambda_{2}$ were tested, specifically 0, 0.25, 0.33 corresponding to one third, 0.5, and 0.67. A total of 100 images with clearly identifiable salient objects were selected from the augmented KU_PCP dataset, consisting of 50 RoT images and 50 Center images. Evaluation metrics comprised objective indicators, including the average values of $A_{c}$ and *A*, together with subjective validation, in which volunteers assessed aesthetic quality on a five-point scale, with 1 indicating poor and 5 indicating excellent.

Table 5 presents the results. $\lambda_{2}\;$= 0.33 (1/3) achieved the highest average $A_{c}$ (0.83 ± 0.07) and subjective aesthetic score (4.2 ± 0.2): $\lambda_{2}\;$= 0 (only $A_{dis}$ considered) led to low $A_{c}$ (0.58 ± 0.09) due to axis misalignment with rule lines, while higher $\lambda_{2}$ values (0.5, 0.67) overemphasized $A_{pos}$ and neglected $A_{dis}$, causing the salient object’s center to deviate from key intersections. Thus, $\lambda_{2}\;$= 1/3 is verified as optimal for prioritizing rule alignment while retaining distance constraints.

### 3.5. Composition Optimization Results

#### 3.5.1. Composition Optimization Results Based on Main Semantic Line

This section presents the composition optimization results based on main semantic line, as shown in Table 6. The first three rows display the optimization results for linear compositions, and the following three rows for symmetric compositions. The first column shows the original images, and the second column shows the images after semantic segmentation. The third column outputs from the composition category determination network, which lists, from top to bottom: Linear, Symmetric, RoT, and Center. The fourth column presents the optimized segmentation results, and the fifth column displays the images optimized by the generative adversarial network.

Table 7 details the changes in IACS evaluation during the image optimization process as shown in Table 6. The first column displays the position of the main semantic line without any composition optimization $P_{s}$, which is the image’s initial state. The second column shows the optimal position of the main semantic line $P_{c}$, as determined by maximizing the compositional aesthetic evaluation function $A_{c}\left(I\right)$, which adjusts the main semantic line to the rule-of-thirds or the center line. The third column presents the final optimized position of the main semantic line $P_{o}$, taking into account both compositional aesthetics and image similarity.

Regarding the evaluation metrics, $A_{c}$ represents the compositional aesthetic evaluation, measuring the aesthetic quality of the image composition; $A_{s}$ represents the similarity evaluation, assessing how similar the optimized image is to the original image; and A as a comprehensive evaluation, is a weighted average of $A_{c}$ and $A_{s}$, providing a comprehensive score that considers both compositional aesthetics and similarity.

According to Table 6, the optimization process brings the main semantic lines closer to the rule-of-thirds or center line, resulting in a more harmonious overall composition. This optimization utilizes a semantic segmentation network that can precisely identify and divide different semantic regions within the image, and the network generates corresponding semantic segmentation result images. The composition category determination network outputs the category of the image’s composition, providing essential references for subsequent compositional optimization. The generative adversarial network outputs the optimized result images. The optimized segmentation results were further processed using DeepSIM to generate the final optimized images. To illustrate the necessity of this step, we conducted a comparative analysis of the images before and after GAN processing. As presented in Table 6, the “Optimized segmentation result” in the fourth column has already improved the compositional arrangement, while the “Optimized result” generated by the GAN in the fifth column further enhances visual realism by smoothing boundaries and preserving fine texture details. This indicates that the GAN not only retains the optimized composition but also enhances perceptual quality, resulting in outputs that are more natural and aesthetically consistent.

According to Table 7, it can be observed that the initial aesthetic evaluation of the composition $A_{c}$ under the initial state $P_{s}$ does not reach a high level, indicating that there is room for improvement in the image composition. For example, the $A_{c}$ of the image in the first row in the $\;P_{s}$ state is only 0.38, but after optimization, it rises to 0.85.

In the second column, when the main semantic line is moved to the optimal composition position $P_{c}$, the aesthetic evaluation $A_{c}$ is enhanced. For example, the image in the first row reaches an $A_{c}$ of 1 at the $P_{s}$ position because the main semantic line is precisely moved to the optimal location. However, such adjustments might reduce the similarity to the original content, causing the similarity evaluation $A_{s}$ to drop, as seen in the first row where the $A_{s}$ decreases to 0.39.

In the third column, when the main semantic line is moved to its final optimized position $P_{o}$ after adjustments, the comprehensive evaluation A is higher than at the original position $P_{s}$ and optimal composition position $P_{c}$. This indicates that the compositional optimization indeed helps enhance the overall aesthetic quality of the image. For example, the comprehensive evaluation A of the image in the first row at the $P_{o}$ position is 0.80, exceeding the $P_{c}$ position’s 0.69 and the $P_{s}$ position’s 0.70, demonstrating the positive effects of compositional optimization.

#### 3.5.2. Composition Optimization Results Based on Salient Object

This section presents the composition optimization results based on salient object, as shown in Table 8. The first three rows display the optimization results for linear compositions, and the following three rows for symmetric compositions. The first column shows the original images, and the second column shows the images after semantic segmentation. The third column outputs from the composition category determination network, which lists, from top to bottom: Linear, Symmetric, RoT, and Center. The fourth column presents the optimized segmentation results, and the fifth column displays the images optimized by the generative adversarial network.

Table 9 details the changes in IACS evaluation during the image optimization process as shown in Table 8. The first column displays the position of the salient object without any composition optimization $P_{s}$, which is the image’s initial state. The second column shows the optimal position of the salient object $P_{c}$, as determined by maximizing the compositional aesthetic evaluation function $A_{c}\left(I\right)$, which adjusts the salient object to the rule-of-thirds or the center line. The third column presents the final optimized position of the salient object $P_{o}$, taking into account both compositional aesthetics and image similarity.

Regarding the evaluation metrics, $A_{c}$ represents the compositional aesthetic evaluation, measuring the aesthetic quality of the image composition; $A_{s}$ represents the similarity evaluation, assessing how similar the optimized image is to the original image; and A as a comprehensive evaluation, is a weighted average of $A_{c}$ and $A_{s}$, providing a comprehensive score that considers both compositional aesthetics and similarity.

According to Table 8, the optimization process brings the salient object closer to the rule-of-thirds or center line, resulting in a more harmonious overall composition. This optimization utilizes a semantic segmentation network that can precisely identify and divide different semantic regions within the image, and the network generates corresponding semantic segmentation result images. The composition category determination network outputs the category of the image’s composition, providing essential references for subsequent compositional optimization. The generative adversarial network outputs the optimized result images.

According to Table 9, it can be observed that the initial aesthetic evaluation of the composition $A_{c}$ under the initial state $P_{s}$ does not reach a high level, indicating that there is room for improvement in the image composition. For example, the $A_{c}$ of the image in the first row in the $\;P_{s}$ state is only 0.40, but after optimization, it rises to 0.87.

In the second column, when the salient object is moved to the optimal composition position $P_{c}$, the aesthetic evaluation $A_{c}$ is enhanced. For example, the image in the first row reaches an $A_{c}$ of 1 at the $P_{s}$ position because the salient object is precisely moved to the optimal location. However, such adjustments might reduce the similarity to the original content, causing the similarity evaluation $A_{s}$ to drop, as seen in the first row where the $A_{s}$ decreases to 0.48.

In the third column, when the salient object is moved to its final optimized position $P_{o}$ after adjustments, the comprehensive evaluation A is higher than at the original position $P_{s}$ and optimal composition position $P_{c}$. This indicates that the compositional optimization indeed helps enhance the overall aesthetic quality of the image. For example, the comprehensive evaluation A of the image in the first row at the $P_{o}$ position is 0.80, exceeding the $P_{c}$ position’s 0.70 and the $P_{s}$ position’s 0.74, demonstrating the positive effects of compositional optimization.

This leads to a crucial insight: even when the main semantic line or salient object is repositioned to an optimal location within the composition, its aesthetic evaluation still falls short compared to when a balance between composition and similarity is achieved. This finding emphasizes the importance of striking a balance between aesthetics and similarity during the optimization of compositions to attain the best visual outcomes.

### 3.6. Comparative Experiments

**Figure 7:**
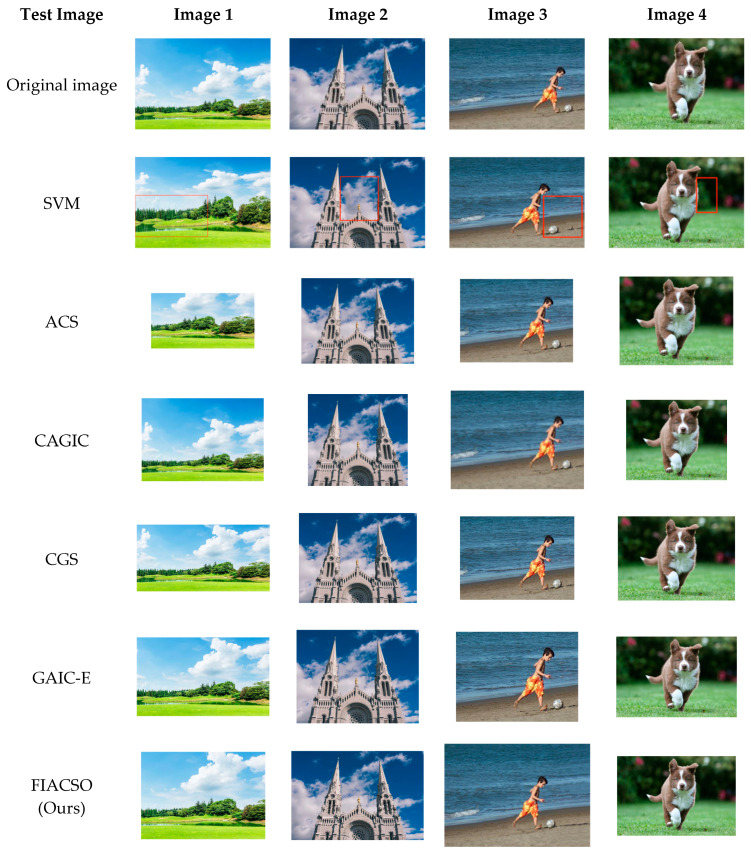
Comparison of experimental results of FIACSO with SVM, CAGIC, ACS, CAGIC, CGS and GAIC-E (red boxes highlight the failure areas).

To validate the effectiveness of the proposed method, we compared it with several previously released approaches for which source code or executables are publicly available: SVM [33], ACS [34], CAGIC [35], CGS [36], and GAIC-E [37], and the comparative results are shown in Figure 7.

Figure 7 displays optimized results of all methods for the four test images, with the original images as the baseline. SVM enhances composition via simple rule-based stretching but fails to protect texture integrity. For example, in the third image of Figure 7, SVM introduces distortion in texture-rich regions and requires manual interaction to reposition salient objects, limiting practicality.

Cropping-based methods ACS, CAGIC, CGS and GAIC-E adjust composition by cropping, yet they share common limitations. First, the loss of background context information occurs. For instance, in the first image of Figure 7, the details of distant scenes are removed. Second, element ratio imbalance arises. Taking the second image of Figure 7 as an example, the horizontal range is narrowed, which impairs visual harmony.

FIACSO (Ours) utilizes Seam Carving and GAN-based refinement (DeepSIM). Seam Carving avoids high-energy content regions, and GAN-based refinement preserves fine details, allowing FIACSO to retain complete salient objects and contextual details while aligning with composition rules. For instance, in the second image of Figure 7, FIACSO optimizes the linear semantic line to the rule of thirds without distorting the landscape’s horizontal balance. In the fourth image of Figure 7, it preserves background context while enhancing the salient object’s positioning.

#### Subjective Evaluation Across Multiple Optimization Methods

To provide a rigorous comparison between FIACSO and established optimization techniques, we conducted a subjective evaluation experiment involving six methods: SVM, ACS, CAGIC, CGS, GAIC-E, and FIACSO. A total of 100 original images were randomly sampled from the KU_PCP dataset, with 25 images selected from each of the four composition categories, namely Linear, Symmetric, RoT, and Center. Each image was optimized by the six methods, producing 600 processed images in total.

Thirty participants took part in the evaluation, including 15 individuals with professional backgrounds in photography or design and 15 individuals without such training. All participants were asked to assess each image along three dimensions of aesthetic evaluation: overall aesthetic appeal, compositional harmony, and content integrity. Ratings were collected on a five-point Likert scale, where a score of one denoted very poor quality and a score of five denoted excellent quality.

Before conducting the main analysis, normality tests were applied to the three variables. Both the Shapiro–Wilk and Kolmogorov–Smirnov tests indicated significant departures from a normal distribution, with all p values less than 0.001, as shown in Table 10. This finding confirmed the need for non-parametric analysis. The descriptive statistics also revealed that the overall mean scores across methods were around 3.25, suggesting a moderate baseline aesthetic evaluation when no distinction among methods was made.

The Kruskal–Wallis test was applied to evaluate whether significant differences existed among the six methods. Table 11 provides the detailed results. All three evaluation dimensions showed statistically significant group differences with chi-square statistics above 5700 and p values less than 0.001. Importantly, the median values indicate that FIACSO achieved higher subjective ratings than the other methods. For overall aesthetic appeal, methods 1 to 5 all yielded a median score of 3, whereas FIACSO reached a median of 5. For compositional harmony and content integrity, methods 1 to 5 also remained at a median of 3, while FIACSO obtained a median of 4. Moreover, the within-group dispersion of FIACSO was consistently lower, with a standard deviation of 0.50 compared to approximately 0.81 for the competing approaches, reflecting more stable ratings across participants.

To further explore where the differences lay, post hoc pairwise comparisons were conducted using the Mann–Whitney U test. Table 12 reports the results for comparisons between FIACSO and each of the other five methods. In all cases, FIACSO obtained significantly higher scores with p values less than 0.001. The effect sizes were larger than 2.0 according to Cohen’s d, representing very large differences in practical terms. By contrast, comparisons among the five conventional methods did not reveal consistent or substantial differences, and their effect sizes were negligible.

This multi-method evaluation provides robust evidence that FIACSO consistently outperforms existing optimization approaches in terms of subjective image aesthetics. The combination of higher median scores, reduced rating variability, and very large effect sizes across all three dimensions demonstrates that FIACSO not only achieves statistical significance but also delivers practically meaningful improvements. The findings highlight its effectiveness in generating visually appealing images while maintaining compositional balance and content integrity, thereby aligning computational optimization with human perceptual judgment.

## 4. Limitations

**Figure 8:**
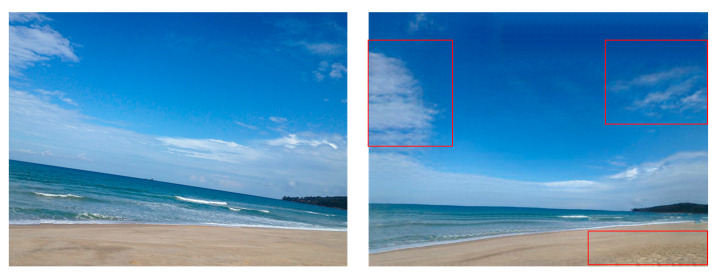
Failure case in severe tilting conditions (red boxes highlight the failure areas).

While the proposed framework demonstrates effectiveness in aesthetics-guided image composition optimization, it still has several limitations. First, the quantitative metrics used in this work, including IACS and SSIM, are designed to approximate human aesthetic judgment but cannot fully reflect subjective preferences influenced by personal and cultural differences. Second, the current method is primarily validated on landscape images with clear semantic structures, and its generalizability to other types of imagery has not yet been verified. Third, when handling images with severe tilting, including those with angles beyond 30 degrees, although the content-aware rotation can properly straighten dominant semantic lines without distorting local textures, the subsequent GAN-based generation process results in texture loss. This issue is particularly noticeable in natural elements like dunes, where fine-grained texture details, including subtle undulations and grainy patterns on dune surfaces, are partially or completely lost, resulting in a smoother, less realistic visual effect, as illustrated in Figure 8.

## 5. Conclusions

In this paper, a method called IACS is proposed, which integrates image composition and similarity. This method uses a unified function to balance the aesthetics of composition and image similarity, and adjusts their influence through parameters. In evaluating the composition aesthetics, it considers the distances between the main semantic line or salient object and the nearest rule-of-thirds line or central line. For images featuring prominent semantic lines, a modified Hough transform is utilized to detect the main semantic line. Similarly, for images containing salient objects, a salient object detection method based on LCSF is applied to determine the salient object region. In evaluating similarity to the original image, edge similarity (measured by the Canny operator) is combined with the SSIM for calculation. Furthermore, a framework of image aesthetic evaluation with composition and similarity-based optimization (FIACSO) is proposed. After categorizing the composition of an image, the framework uses a semantic segmentation network to segment the image (and thus obtain composition information) and applies the IACS method for optimization. Ultimately, it uses a GAN to generate an optimized image that adheres to compositional rules and closely resembles the original image.

Experimental and comparative results show that FIACSO enhances image composition, minimizes visual distortion, and preserves the original content to a great extent---thus optimizing image processing and elevating the image’s aesthetic value. It exhibits high accuracy and generalization capabilities in image composition optimization. Furthermore, subjective evaluations involving human participants demonstrate that FIACSO significantly outperforms existing methods in terms of overall aesthetics, compositional harmony, and content integrity, validating its effectiveness from a human-centric perspective.

Beyond empirical performance, the study makes a theoretical contribution by grounding the optimization process in Gestalt visual psychology. By enhancing figure–ground separation, maintaining structural continuity, and aligning with the principles of balance and proximity, the framework generates results that align with human perceptual mechanisms. This alignment between computational modeling and psychological theory highlights the robustness and interpretability of the method.

Equally important is the practical value of the framework. In professional contexts, it provides a reliable tool for photographers, designers, and visual curators to refine large-scale image collections with high efficiency and consistency. In digital platforms and online environments, it has the potential to be deployed in content creation, sharing, and recommendation systems, where automated aesthetic assessment and real-time optimization can improve both user experience and visual communication quality.

Nevertheless, the current focus on conventional compositional rules limits its scope. Future research will broaden the optimization strategy by incorporating multimodal visual features—including color harmony, texture, and lighting—as well as semantic dimensions such as object categories and scene context. Additionally, the proposed framework’s quantitative metrics cannot fully capture subjective aesthetic preferences shaped by personal and cultural factors; moreover, it may lose fine-grained texture details when optimizing severely tilted images. These are limitations to be addressed in subsequent work.

## Figures and Tables

**Table 1:** Experimental hardware and software configuration.

Item	Detail
CPU	13th Gen Intel^®^ Core^TM^ i7-13700KF @3.40 GHz
RAM	32 GB
Operating system	Windows 11 64-bit
CUDA	11.8
Python	3.8
PyTorch	1.10.0
PyCharm	2025.1

**Table 2:** The number of images before and after data augmentation.

Composition Category	Before Augmentation	After Augmentation
Linear	758	2274
Symmetric	232	696
RoT	923	2769
Center	750	2250

**Table 3:** Comparative results between CNN and Transformer.

Model Type	Model Name	Image Size	Image Size	Mean F1-Score	Top-1 Acc (%)
CNN	ResNet50	224 × 224	25.56	0.735	71.89
	ResNext50	224 × 224	25.03	0.684	75.42
	MobileNetV2	224 × 224	3.5	0.812	65.87
	MobileNetV3	224 × 224	5.48	0.710	71.35
	ConvNeXt	224 × 224	87.57	0.811	83.58
Transformer	ViT-B/16	224 × 224	103.19	0.731	74.64
	ViT-L/16	224 × 224	326.74	0.772	78.42
	Swin-Base	224 × 224	87.76	0.867	**86.96**
	Swin-Large	224 × 224	228.57	0.814	84.90
	MobileViT-XXS	224 × 224	1.27	0.812	80.14
	MobileViT-XXS	224 × 224	2.32	0.791	79.23
	MobileViT-S	224 × 224	5.58	0.801	81.35

Note: Best scores are bolded.

**Table 4:** Sensitivity Analysis Results for *λ*_1_.

*λ*_1_	$\mathrm{Average}\;A_{c}$ (±SD)	$\mathrm{Average}\;A_{s}$ (±SD)	Average A (±SD)	Subjective Preference Rate (%)
0.1	0.41 ± 0.08	0.92 ± 0.05	0.67 ± 0.06	28 ± 5
0.3	0.63 ± 0.07	0.85 ± 0.06	0.71 ± 0.05	55 ± 7
0.5	**0.82 ± 0.06**	**0.74 ± 0.07**	**0.80 ± 0.04**	**82 ± 4**
0.7	0.91 ± 0.05	0.58 ± 0.08	0.77 ± 0.05	63 ± 6
0.9	0.96 ± 0.04	0.40 ± 0.09	0.68 ± 0.07	35 ± 5

Note: Best scores are bolded.

**Table 5:** Sensitivity Analysis Results for *λ*_2_.

*λ*_2_	$\mathrm{Average}\;A_{c}$ (±SD)	Average A (±SD)	Average Subjective Aesthetic Score (±SD)
0	0.58 ± 0.09	0.65 ± 0.07	2.8 ± 0.4
0.25	0.69 ± 0.08	0.73 ± 0.06	3.5 ± 0.3
0.33	**0.83 ± 0.07**	**0.81 ± 0.05**	**4.2 ± 0.2**
0.5	0.78 ± 0.08	0.77 ± 0.06	3.8 ± 0.3
0.67	0.65 ± 0.09	0.70 ± 0.07	3.2 ± 0.4

Note: Best scores are bolded.

**Table 6:** The composition optimization results based on main semantic lines.

Original Image	Semantic Segmentation Result	Classification Result	Optimized Segmentation Result	Optimized Result
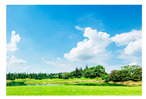	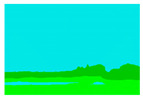	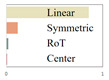	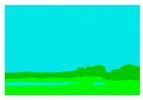	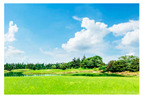
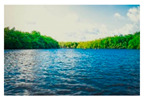	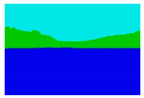	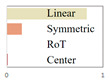	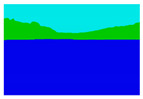	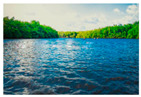
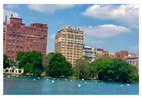	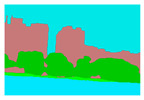	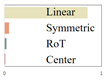	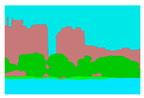	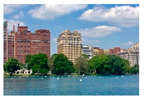
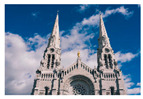	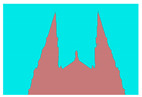	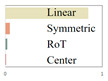	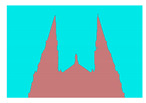	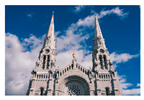
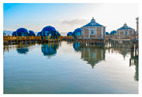	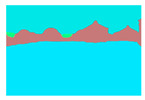	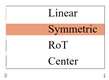	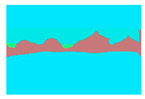	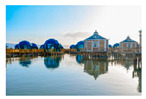
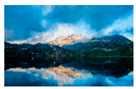	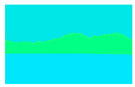	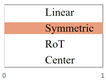	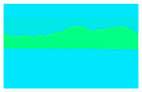	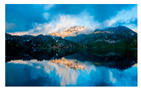

Note: The “Classification Result” column lists the four composition classes—Linear, Symmetric, RoT, Center—in top-to-bottom order. A “1” denotes the model’s predicted composition class for the input image, and “0” denotes classes that do not match the image’s composition.

**Table 7:** The IACS evaluation of the image optimization process with main semantic line.

$P_{s}$			$P_{c}$			$P_{o}$		
$A_{c}$	$A_{s}$	A	$A_{c}$	$A_{s}$	A	$A_{c}$	$A_{s}$	A
0.38	1	0.69	1	0.39	0.70	0.85	0.74	**0.80**
0.40	1	0.72	1	0.48	0.74	0.87	0.75	**0.8** **1**
0.37	1	0.69	1	0.47	0.74	0.88	0.74	**0.8** **1**
0.52	1	0.76	1	0.55	0.79	0.90	0.77	**0.8** **4**
0.54	1	0.77	1	0.57	0.78	0.87	0.79	**0.8** **3**
0.55	1	0.78	1	0.58	0.79	0.89	0.78	**0.8** **4**

Note: Best scores are bolded.

**Table 8:** The composition optimization results based on salient object.

Original Image	Segmentation Result	Classification Result	Optimized Segmentation Result	Optimized Result
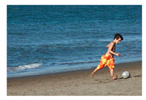	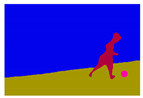	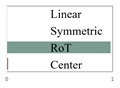	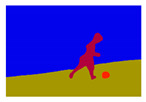	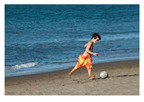
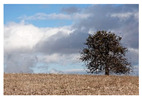	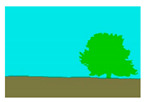	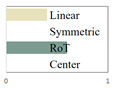	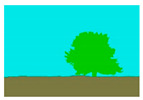	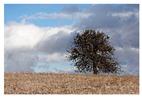
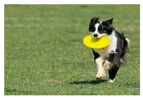	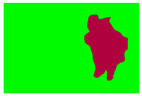	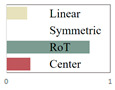	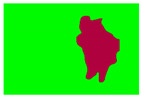	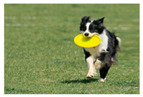
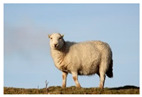	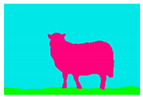	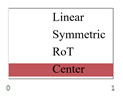	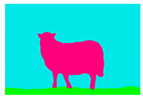	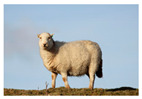
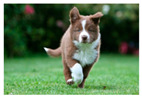	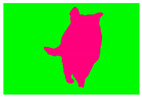	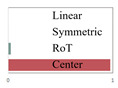	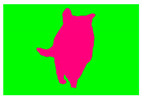	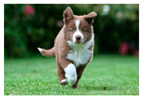
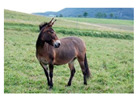	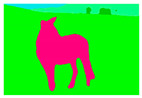	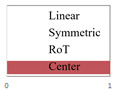	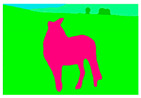	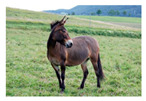

Note: The “Classification Result” column lists the four composition classes—Linear, Symmetric, RoT, Center—in top-to-bottom order. A “1” denotes the model’s predicted composition class for the input image, and “0” denotes classes that do not match the image’s composition.

**Table 9:** The IACS evaluation of the image optimization process with salient object.

$P_{s}$			$P_{c}$			$P_{o}$		
$A_{c}$	$A_{s}$	A	$A_{c}$	$A_{s}$	A	$A_{c}$	$A_{s}$	A
0.40	1	0.70	1	0.48	0.74	0.87	0.72	**0.80**
0.42	1	0.71	1	0.52	0.76	0.86	0.76	**0.8** **2**
0.49	1	0.75	1	0.51	0.76	0.87	0.74	**0.8** **1**
0.50	1	0.75	1	0.54	0.77	0.88	0.79	**0.8** **4**
0.48	1	0.74	1	0.57	0.79	0.87	0.78	**0.8** **3**
0.46	1	0.73	1	0.55	0.78	0.89	0.77	**0.8** **3**

Note: Best scores are bolded.

**Table 10:** Normality test results for subjective evaluation data.

Variable	N	Mean	SD	Skewness	Kurtosis	Shapiro–Wilk p	Kolmogorov–Smirnov p
Overall aesthetic	18,000	3.256	0.956	0.055	–1.098	0.000 ***	0.000 ***
Compositional harmony	18,000	3.246	0.952	0.068	–1.089	0.000 ***	0.000 ***
Content integrity	18,000	3.244	0.951	0.065	–1.094	0.000 ***	0.000 ***

Note: *** denotes significance levels of 1%.

**Table 11:** Kruskal–Wallis test results for subjective evaluation across the optimization methods.

Analysis Item	Group	Sample Size	Median	SD	$\chi^{2}$	p	Cohen’s f
Overall aesthetic	1	3000	3	0.82	5754.524	0.000 ***	0.011
	2	3000	3	0.816			
	3	3000	3	0.815			
	4	3000	3	0.816			
	5	3000	3	0.82			
	6	3000	5	0.5			
	Total	18,000	3	0.956			
Compositional harmony	1	3000	3	0.81	5744.216	0.000 ***	0.011
	2	3000	3	0.82			
	3	3000	3	0.813			
	4	3000	3	0.821			
	5	3000	3	0.805			
	6	3000	4	0.5			
	Total	18,000	3	0.952			
Content integrity	1	3000	3	0.814	5705.669	0.000 ***	0.011
	2	3000	3	0.819			
	3	3000	3	0.817			
	4	3000	3	0.806			
	5	3000	3	0.817			
	6	3000	4	0.5			
	Total	18,000	3	0.951			

Note: *** denotes significance levels of 1%.

**Table 12:** Pairwise Mann–Whitney U test results between the optimization methods.

Comparison		Median		U Statistic	p	Cohen’s *d*
Group A	Group B	Group A	Group B			
Overall aesthetic_SVM	Overall aesthetic_ACS	3	3	4,319,498.5	0.009 ***	0.074
Overall aesthetic_SVM	Overall aesthetic_CAGIC	3	3	4,376,036.5	0.100	0.051
Overall aesthetic_SVM	Overall aesthetic_CGS	3	3	4,360,197.5	0.054 *	0.057
Overall aesthetic_SVM	Overall aesthetic_GAIC-E	3	3	4,444,223.5	0.756	0.023
Overall aesthetic_SVM	Overall aesthetic_FIACSO	3	5	711,228.5	0.000 ***	2.265
Overall aesthetic_ACS	Overall aesthetic_CAGIC	3	3	4,557,187.5	0.732	0.023
Overall aesthetic_ACS	Overall aesthetic_CGS	3	3	4,541,099.5	1.032	0.017
Overall aesthetic_ACS	Overall aesthetic_GAIC-E	3	3	4,624,577.5	0.098 *	0.051
Overall aesthetic_ACS	Overall aesthetic_FIACSO	3	5	771,539.5	0.000 ***	2.183
Overall aesthetic_CAGIC	Overall aesthetic_CGS	3	3	4,483,933	1.599	0.007
Overall aesthetic_CAGIC	Overall aesthetic_GAIC-E	3	3	4,567,885	0.566	0.028
Overall aesthetic_CAGIC	Overall aesthetic_FIACSO	3	5	747,268	0.000 ***	2.213
Overall aesthetic_CGS	Overall aesthetic_GAIC-E	3	3	4,583,788	0.371	0.034
Overall aesthetic_CGS	Overall aesthetic_FIACSO	3	5	754,623	0.000 ***	2.204
Overall aesthetic_GAIC-E	Overall aesthetic_FIACSO	3	5	731,087	0.000 ***	2.237
Compositional harmony_SVM	Compositional harmony_ACS	3	3	4,449,433.5	0.848	0.021
Compositional harmony_SVM	Compositional harmony_CAGIC	3	3	4,411,494	0.323	0.036
Compositional harmony_SVM	Compositional harmony_CGS	3	3	4,448,442	0.830	0.021
Compositional harmony_SVM	Compositional harmony_GAIC-E	3	3	4,417,972.5	0.389	0.033
Compositional harmony_SVM	Compositional harmony_FIACSO	3	4	716,578.5	0.000 ***	2.258
Compositional harmony_ACS	Compositional harmony_CAGIC	3	3	4,463,029.5	1.118	0.015
Compositional harmony_ACS	Compositional harmony_CGS	3	3	4,499,008.5	1.975	0
Compositional harmony_ACS	Compositional harmony_GAIC-E	3	3	4,469,865	1.268	0.012
Compositional harmony_ACS	Compositional harmony_FIACSO	3	4	755,007	0.000 ***	2.212
Compositional harmony_CAGIC	Compositional harmony_CGS	3	3	4,535,960	1.139	0.015
Compositional harmony_CAGIC	Compositional harmony_GAIC-E	3	3	4,507,227.5	1.818	0.003
Compositional harmony_CAGIC	Compositional harmony_FIACSO	3	4	754,253.5	0.000 ***	2.209
Compositional harmony_CGS	Compositional harmony_GAIC-E	3	3	4,470,882.5	1.291	0.012
Compositional harmony_CGS	Compositional harmony_FIACSO	3	4	755,760.5	0.000 ***	2.211
Compositional harmony_GAIC-E	Compositional harmony_FIACSO	3	4	738,430	0.000 ***	2.227
Content integrity_SVM	Content integrity_ACS	3	3	4,592,944.5	0.283	0.038
Content integrity_SVM	Content integrity_CAGIC	3	3	4,536,948	1.118	0.015
Content integrity_SVM	Content integrity_CGS	3	3	4,657,947.5	0.025 **	0.065
Content integrity_SVM	Content integrity_GAIC-E	3	3	4,550,940.5	0.841	0.021
Content integrity_SVM	Content integrity_FIACSO	3	4	783,360	0.000 ***	2.177
Content integrity_ACS	Content integrity_CAGIC	3	3	4,444,056	0.753	0.023
Content integrity_ACS	Content integrity_CGS	3	3	4,563,176	0.636	0.026
Content integrity_ACS	Content integrity_GAIC-E	3	3	4,458,047	1.014	0.017
Content integrity_ACS	Content integrity_FIACSO	3	4	758,016	0.000 ***	2.212
Content integrity_CAGIC	Content integrity_CGS	3	3	4,620,184	0.115	0.049
Content integrity_CAGIC	Content integrity_GAIC-E	3	3	4,513,984	1.650	0.006
Content integrity_CAGIC	Content integrity_FIACSO	3	4	774,144	0.000 ***	2.19
Content integrity_CGS	Content integrity_GAIC-E	3	3	4,394,081	0.188	0.044
Content integrity_CGS	Content integrity_FIACSO	3	4	710,400	0.000 ***	2.269
Content integrity_GAIC-E	Content integrity_FIACSO	3	4	770,304	0.000 ***	2.195

Note: ***, **, * denote significance levels of 1%, 5%, and 10%, respectively.

## Data Availability

The code for our proposed framework FIACSO and dataset used in the experiments can be found on GitHub: https://github.com/zafucslab/FIACSO (accessed on 17 June 2024).
